# Joint association of sleep duration and physical activity with constipation: a cross-sectional study

**DOI:** 10.7189/jogh.16.04178

**Published:** 2026-06-19

**Authors:** Xiaohui Zhang, Zhantong Wang, Huan Zhang, Weijia Dou, Zhenxiong Liu, Lei Shang

**Affiliations:** 1 Department of Health Statistics, School of Preventive Medicine, Fourth Military Medical University, Xi'an, Shaanxi, China; 2 Department of Gastroenterology, Tangdu Hospital, Fourth Military Medical University, Xi'an, Shaanxi, China; 3 Xijing 986 Hospital Department, Fourth Military Medical University, Xi'an, Shaanxi, China; 4 Department of Gastroenterology, The 961th Hospital of Joint Logistics Support Force of PLA, Qiqihar, China

## Abstract

**Background:**

Sleep duration and physical activity (PA) may play a pivotal role in gastrointestinal functions. It remains unclear how they function in constipation. This study aims to assess the independent and combined associations of sleep duration and PA with the risk of constipation, using data from the National Health and Nutrition Examination Survey (NHANES) 2007–2010.

**Methods:**

A total of 5590 (unweighted data) participants were included in the analysis. Sleep duration was categorised as insufficient (<7 hours/d), sufficient (7–8h/d), and excessive (>8 hours/d). Physical activity was converted to metabolic equivalent (MET) minutes of moderate to vigorous PA per week, classified as inactive (<600 MET-minutes/week) and active (≥600 MET-minutes/week). Constipation was defined based on stool consistency using the Bristol Stool Form Scale. Multivariable logistic regression models were used to estimate the odds ratios (ORs) for constipation.

**Results:**

In multivariable-adjusted models, sufficient sleep duration and active PA was associated with reduced ORs of constipation. Subgroup analysis found this association was more pronounced in male population older than or equal to 60 (sufficient sleep (OR = 0.14; 95% CI = 0.02–0.75, *P* = 0.047) and active PA (OR = 0.27; 95% CI = 0.08–0.93, *P* = 0.050)), but not younger adults and female participants. Furthermore, active PA was always associated with a lower risk of constipation, no matter how long the sleep duration was (insufficient sleep (OR = 0.13; 95% CI = 0.03–0.57, *P* = 0.030), sufficient sleep (OR = 0.04; 95% CI = 0.01–0.21, *P* = 0.006), and excessive sleep (OR = 0.02; 95% CI = 0.00–0.42, *P* = 0.038)).

**Conclusions:**

Sufficient sleep as well as active PA were associated with lower risk of constipation, independently or jointly. This finding was more pronounced in male population older than or equal to 60, but not younger adults and female participants.

Constipation is a common gastroenterology clinic symptom, featuring as decreased colonic motility or difficulty with the defecation. Its prevalence rates ranging from 2 to 39% [1–5], which may be attributed to both differences in populations studied and the criteria used to define constipation [6,7]. Constipation can be influenced by a wide variety of factors like diet, psychology, medication, bowel habits, sleep habit, physical activity, *etc*. The treatment effects for constipation vary greatly among different patients, and often it can be recurrent and chronic. Therefore, it is particularly important to identify modifiable risk factors for constipation to prevent from such clinical symptoms.

Limited number of researches have explored the relationship between sleep duration and constipation [8–15]. Their conclusions varied greatly. Huang’s group found long sleep duration was associated with a lower constipation risk (OR = 0.725; 95% CI = 0.553–0.952) [15], while others thought shorter and longer sleep durations were both associated with a higher likelihood of constipation than an intermediate duration (6.5–7.4 hours/d) [8–14]. Besides, Hou’s group demonstrated there was no statistically significant association between sleep duration and functional constipation. What’s more, Dong Tai et al. declared men with short sleep duration correlated with increased odds for constipation, while women with long sleep duration linked to the higher constipation risk [12]. On the other hand, PA improves bowel function by various means like enhancing gastrointestinal motility, reducing transit time, improving gut flora, and so on [16,17]. Most previous researches revealed constipation was associated with insufficient exercise [18] and Liu’s group suggested engaging in regular PA, with 20–30 minutes per session, at least five days a week, effectively reduced the likelihood of constipation [19]. Hofman’ group found that higher level of PA was associated with a decreased risk of constipation in preschool children [20], however, others believed physical activity appears to be unrelated to the risk of constipation in adolescent or employed adults [21,22]. Thus, the relationship between sleep duration, PA and constipation remains unclear. Factors like different sleep duration criteria, PA criteria, constipation definition, and other adjustable risk factors play a pivotal role in these variations, among which sex and age were the most mentioned.

Sleep duration and PA have been widely reported to share a bidirectional relationship. A meta-analysis reviewed a weak negative association between moderate-to-vigorous PA level and sleep duration (r = −0.02; 95% CI = −0.16–0.12, *P* = 0.760) [23] and another randomised controlled trail found that PA intervention could significantly improve sleep efficacy, sleep onset latency, and sleep duration in children with autism spectrum disorder [24]. Evidences showed that joint association between sleep and PA had an influence on cognitive ageing [25], cardiovascular disease and cancer mortality risk [26], *etc*. However, studies on whether sleep duration and PA jointly affect constipation is scarce. Answering this question will help us better understand how to coordinate sleep duration and PA for constipation prevention.

In the current study, we aimed to investigate the independent associations of PA and sleep duration with constipation, as well as how these factors interact to play a role in constipation. We used National Health and Nutrition Examination Survey (NHANES) 2007–2010, considering its large sample size, high-quality and representative data. What’s more, we explored the dose-response relationship between these factors and constipation. Applicability in different subgroup population was also researched. Here, we assumed that PA and sleep duration might help improve constipation, jointly or independently, which might vary among people of different age and sex.

**Adherence to JoGH’s Guidelines for Reporting Analyses of Big Data Repositories Open to the Public (GRABDROP)**

This secondary analysis was conducted and reported in accordance with the JoGH’s Guidelines for Reporting Analyses of Big Data Repositories Open to the Public (GRABDROP) [27] (Table S1 in the **Online Supplementary Document**).

## METHODS

### Data source

This is a cross-sectional study. Data used in this study was obtained from NHANES, where health and nutritional status of a nationally representative sample of the US population was evaluated by using a multistage, stratified, clustered probabilistic design. Specifically, the sampling process involves selecting primary sampling units (counties), followed by segments (city blocks), households, and finally individual participants. As certain groups (*e.g*. older adults, low-income individuals) are oversampled to increase statistical reliability, sampling weights were applied in all analyses to account for the unequal probability of selection and non-response, and to adjust for the complex survey structure. This study extracted demographic data, questionnaire interviews, and physical examinations from the two consecutive NHANES cycles (2007–2008 and 2009–2010). The NHANES study protocol was approved by the National Centre for Health Statistics Ethical Review Board and written informed consent was obtained from all participants. All procedures performed in this study were in accordance with the 1964 Helsinki Declaration and its later amendments or comparable ethical standards.

### Study population

**Figure 1:**
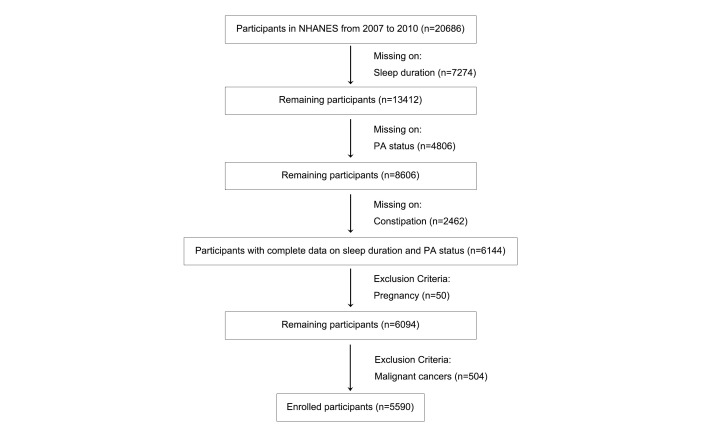
Flowchart of participant enrolment. A total of 20 686 participants in NHANES from 2007 to 2010 were enrolled. Samples missing data on sleep duration (n = 7274), PA status (n = 4806), and constipation (n = 2462) were excluded, and 6144 participants were left. Fifty pregnant participants and 504 malignant cancer patients were also excluded, and 5590 subjects were enrolled in the final analysis.

A total of 20 686 participants in NHANES from 2007 to 2010 were enrolled. After excluding samples with missing data on sleep duration (n = 7274), PA status (n = 4806), and constipation (n = 2462), 6144 participants with complete data were enrolled. Among them, 50 pregnant participants and 504 malignant cancer patients were also excluded, and 5590 subjects were included in the final analysis (**Figure 1**).

### Measurement of sleep duration

Sleep duration collected by NHANES was defined as the response to the question: How much sleep do you get (hours)? Specifically, it focused on the sleep duration participants usually get at night on weekdays or workdays, which was related to constipation. Their response was divided into three groups. Those sleeping less than seven hours were thought to have an insufficient sleep duration, more than eight hours were excessive sleep duration, and the others were sufficient [28].

### Measurement of PA status

Physical activity score was also collected based on the existing NHANES data, according to the Global Physical Activity Questionnaire (GPAQ) Analysis Guide (https://www.who.int/docs/default-source/ncds/ncd-surveillance/gpaq-analysis-guide.pdf). The Global Physical Activity Questionnaire collects information on physical activity participation in three domains, including leisure-time PA, occupation PA, and transportation PA during the last 30 days. Metabolic equivalent is the ratio of a person's working metabolic rate relative to the resting metabolic rate, and is commonly used to express the intensity of equivalent physical activities. Different PA domain possesses different MET, recommended by NHANES. The Global Physical Activity Questionnaire also collected the days participants spend doing different domains of physical activity in a typical week, and the hours they spend on a typical day. These data are self-reported. Finally, PA score in each domain was calculated as follows: PA score (MET-min/week) = MET × days per weekly × hours per day. And the total PA score was the summary of PA score for each domain. Respondents were classified as active PA, meeting the recommended guidelines for adults (≥600 MET-minutes/week, equivalent to 150-minute/week of moderate-intensity or 75-minute/week of vigorous-intensity physical activity) or as inactive PA, not meeting the recommended guidelines for adults (<600 MET-minutes/week).

### Measurement of constipation

Constipation information was obtained from The Bowel Health Questionnaire (https://wwwn.cdc.gov/Nchs/Data/Nhanes/Public/2007/DataFiles/BHQ_E.htm). Both data collection and analysis were based on existing NHANES data. Participants were shown a card on which there were descriptions of the seven Bristol Stool Form Scale types (BSFS; Type 1-Type 7) and asked: ‘Please look at this card and tell me the number that corresponds with your usual or most common stool type’. Consistent with previous research, constipation was defined as a usual or most common stool type of BSFS Type 1 (separate hard lumps, like nuts) or Type 2 (sausage-like, but lumpy). Healthy control was defined as Type 3 (like a sausage but with cracks in the surface), Type 4 (like a sausage or snake, smooth and soft), or Type 5 (soft blobs with clear-cut edges) [29–31].

### Study covariables

Covariables information in this study was obtained from the original NHANES data and was not collected or generated by the authors. The selection of demographic covariables follows previous similar studies based on NHANES, including sex, age, BMI, race, education level, marriage, PIR, smoking, and drinking. Some demographic covariates, as well as hypertension and diabetes available in NHANES, are considered to be clinically related to constipation based on clinical experience and previous studies. Therefore, these are also included as covariables. In addition, variables from NHANES with a missing rate above 10% were excluded. For example, objective glycaemic and blood pressure measures were considered but not used due to sample constraints. Other factors, like bowel habit, time to fall asleep, circadian clock, and so on were not available in NHANES, thus, can also not be regarded as covariables. Those covariables considered significantly related in univariate analysis were further adjusted in multivariate logistic regression analysis (*P* < 0.05). Demographic information was obtained through a structured questionnaire created by face-to-face interviews implemented by trained staff. Body mass index (BMI) was calculated as weight (kg) divided by height squared (m^2^). Participants who smoked at least 100 cigarettes in their life were defined as smoker. In terms of drinking status, one alcohol-based drink was defined as 12 ounces of beer, four ounces of wine, or one ounce of liquor in NHANES. Non-drinkers were those who had consumed fewer than 12 alcohol-based drinks in the past year or lifetime; former drinkers had consumed at least 12 drinks in their lifetime but not in the past year; and current drinkers had at least 12 drinks in the past year and reported a nonzero number of drinks per week [32]. There were three questions used to determine whether one had diabetes: ‘Did a doctor tell you that you have diabetes?’; ‘Are you currently taking insulin?’; ‘Are you taking diabetic pills to lower blood glucose?’. If any of the three questions was answered ‘Yes’, participants were classified as having diabetes. If all responses were ‘No’, they were classified as not having diabetes. Objective glycaemic and blood pressure measures were considered but not used due to reference on previous studies [33–36], sample constraints, and our aim on exploring the relationship between lifestyle and constipation rather than clinically undiagnosed diabetes or hypertension. Similarly, hypertension was diagnosed based on three questions: ‘Have you ever been told you had high blood pressure?’; ‘Have you been told you had high blood pressure on two or more occasions?’; ‘Are you currently taking prescription medication for hypertension?’ If any of the three questions was answered ‘Yes’, participants were classified as having hypertension. Conversely, if all three questions were answered ‘No’, they were classified as not having hypertension. Depressive symptoms were assessed by the Patient Health Questionnaire-9 items (PHQ-9), which is a 9-item validated, and publicly available depression questionnaire. Total PHQ-9 score ranges from 0 to 27. A total score of 0–4 was identified as ‘no depression’, while ≥5 was identified as ‘depression’. All variables used in the analysis were obtained from the same participants, and data sets were merged via participant ‘Sequence’ exported from NHANES. Some participants had missing covariables information. However, the proportion of missing data was negligible and unlikely to affect the main results of our analysis.

### Statistical analysis

According to the NHANES analytical guidelines, sample weights, clustering, and stratification were employed in the statistical analysis. Characteristics of participants were described using median and interquartile range (IQR) for continuous variables, and frequency and proportion for categorical variables. One-way ANOVA was used for continuous variables comparation, while χ^2^ was used for categorical variables. Weighted logistic regression model was employed to find the associations between sleep duration or PA status with constipation. The restricted cubic spline with three nots was modelled to explore the potential nonlinear correlation between exact sleep duration (hours) or PA score (MET-minutes/week) and constipation. Population attributable fraction (PAF) analysis was used to evaluated the decreased percentage of healthy controls if sufficient sleep and/or active PA were cancelled. Sensitive analysis was used to verify the robustness of our results. Statistical analysis was performed using R studio, version 4.4.1 (Posit PBC, Boston, Massachusetts, USA). R packages including tableone package, gtsummary package, survey package and rms package were employed. *P* value <0.05 (2 tailed) was considered statistically significant. No correction for multiple testing may increase the risk of type I errors.

## RESULTS

### Baseline characteristics

A total of 115.8 million (weighted data) US adults were included in the present study. **Table 1** presented the unweighted sociodemographic characteristics, sleep duration and PA status of the study population, stratified by whether suffering from constipation or not. Among them, 432 (7.7%) were diagnosed with constipation. 2521 (45.1%) were female and 3069 (54.9%) were male. Participants with or without constipation exhibited certain distinguishing features. Participants with constipation were generally younger, more likely to be female, non- Hispanic black, less educated, and less likely to drink. They had lower poverty-to-income ratio (PIR), insufficient or excessive sleep duration, and were engaged in less physical activity. The demographic characteristics were similar between the population included in this study (n = 5590) and the weighted study population (n = 115.8 million) (Table S2 in the **Online Supplementary Document**).

**Table 1:** Unweighted characteristics of participants stratified by constipation status (constipation *vs*. no constipation)

Characteristics*	Constipation		*P*-value
	**No**	**Yes**	
**Overall**	5158 (92.3)	432 (7.7)	
**Sex**			
Female	2241 (43.4)	280 (64.8)	<0.001†
Male	2917 (56.6)	152 (35.2)	
**Age, MD (IQR)**	44.00 (31.00–58.00)	42.00 (29.00–55.00)	0.014†
**BMI**			
Normal	1461 (28.4)	147 (34.1)	0.096
Underweight	70 (1.4)	6 (1.4)	
Overweight	1829 (35.6)	143 (33.2)	
Obese	1778 (34.6)	135 (31.3)	
**Race**			
Mexican American	904 (17.5)	67 (15.5)	0.005†
Non-Hispanic White	2568 (49.8)	187 (43.3)	
Non-Hispanic Black	939 (18.2)	99 (22.9)	
Others	747 (14.5)	79 (18.3)	
**Education level**			
<High school	416 (8.1)	52 (12.0)	<0.001†
High school	1954 (37.9)	191 (44.2)	
>High school	2782 (54.0)	189 (43.8)	
**Marriage**			
Married	2698 (52.3)	204 (47.2)	0.058
Widowed	247 (4.8)	27 (6.2)	
Divorced	521 (10.1)	52 (12.0)	
Living with partner	452 (8.8)	44 (10.2)	
Never married	1064 (20.6)	98 (22.7)	
Separated	174 (3.4)	7 (1.6)	
**PIR**			
<1	1274 (24.7)	137 (31.7)	0.001†
1–3	1947 (37.7)	166 (38.4)	
≥3	1937 (37.6)	129 (29.9)	
**Smoking**			
No	2820 (54.7)	251 (58.1)	0.186
Yes	2337 (45.3)	181 (41.9)	
**Drinking**			
No drink	562 (12.1)	70 (17.5)	<0.001†
Former drinker	614 (13.3)	69 (17.2)	
Current drinker	3453 (74.6)	261 (65.2)	
**Hypertension**			
No	3712 (72.1)	331 (76.8)	0.040†
Yes	1439 (27.9)	100 (23.2)	
**Diabetes**			
No	4714 (91.5)	399 (92.4)	0.588
Yes	439 (8.5)	33 (7.6)	
**Sleep duration**			
Insufficient	2010 (39.0)	213 (49.3)	<0.001†
Sufficient	2844 (55.1)	192 (44.4)	
Excessive	304 (5.9)	27 (6.2)	
**PA status**			
Active	4097 (79.4)	319 (73.8)	0.007†
Inactive	1061 (20.6)	113 (26.2)	

BMI – body mass index, IQR – interquartile range, MD – median, PA – physical activity, PIR – poverty-to-income ratio

*Data shown are n (%) unless otherwise specified.

†*P* < 0.05.

### Association of sleep duration and physical activity with constipation

The association of sleep duration and PA status with constipation were investigated by the weighted logistic regression models. Unadjusted model, where other covariables that could influence the outcome were not accounted for, revealed that compared with people who had insufficient sleep duration, those with sufficient sleep duration had a lower risk of constipation (OR = 0.61; 95% CI = 0.50–0.74, *P* < 0.001), and compared with people who were in inactive PA status, those with active PA status were less likely to be with constipation (OR = 0.70; 95% CI = 0.53–0.95, *P* = 0.022). Diabetes, and hypertension were not found related to constipation risk, therefore, these variables were not included as further adjustments in the multivariate logistic regression models. After fulling adjusting for sex, age, BMI, race, education level, marriage status, PIR, and drinking status, controlling for these potential confounding factors, sufficient sleep duration was found to be independently associated with a lower likelihood of constipation (OR = 0.62; 95% CI = 0.48–0.80, *P* = 0.006), while active PA status was not (OR = 0.82; 95% CI = 0.58–1.16, *P* = 0.298) (**Table 2**).

**Table 2:** Association of sleep duration and PA status with constipation

	Unadjusted model		Adjusted model*	
	**OR (95% CI)**	***P*-value**	**OR (95% CI)**	***P*-value**
**Sleep duration**				
Insufficient	1.00 (Reference)		1.00 (Reference)	
Sufficient	0.61 (0.50–0.74)	<0.001†	0.62 (0.48–0.80)	0.006†
Excessive	0.82 (0.51–1.33)	0.405	0.74 (0.44–1.23)	0.277
**PA status**				
Inactive	1.00 (Reference)		1.00 (Reference)	
Active	0.70 (0.53–0.95)	0.022†	0.82 (0.58–1.16)	0.298

CI – confidence interval, OR – odds ratios, PA – physical activity

*Adjusted model was adjusted for sex, age, body mass index, race, education level, marriage status, PIR and drinking status.

*P < 0.05.

**Figure 2:**
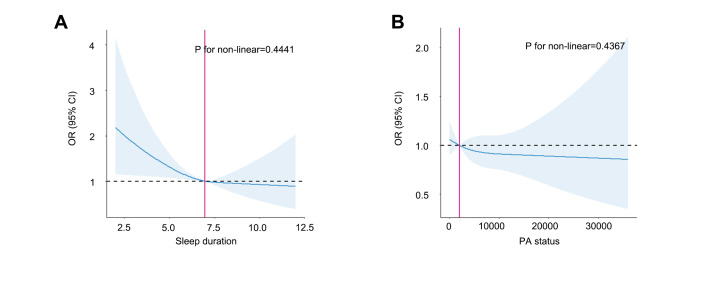
Dose-response associations between sleep duration, and PA with constipation. **Panel A.** Relationship between sleep duration and constipation was L-shaped with a turning point at about seven hours. **Panel B.** Relationship between PA score and constipation was L-shaped with a turning point at about 1847 MET-minutes/week. ORs (95% CI) were calculated using multivariate logistic regression analysis adjusted for sex, race, education level, PIR and drinking status. CI – confidence interval, MET – metabolic equivalent, OR – odds ratios, PA – physical activity.

Moreover, the restricted cubic spline model revealed that sleep duration and PA score were linearly related to constipation outcomes (nonlinear *P* = 0.444 for sleep duration, nonlinear *P* = 0.437 for PA score). We found the L-shaped relationship between sleep duration and constipation with a turning point at about seven hours and the L-shaped relationship between PA score and constipation with a turning point at about 1847 MET-minutes/week (**Figure 2**).

### Joint association of sleep duration and physical activity with constipation

**Figure 3:**
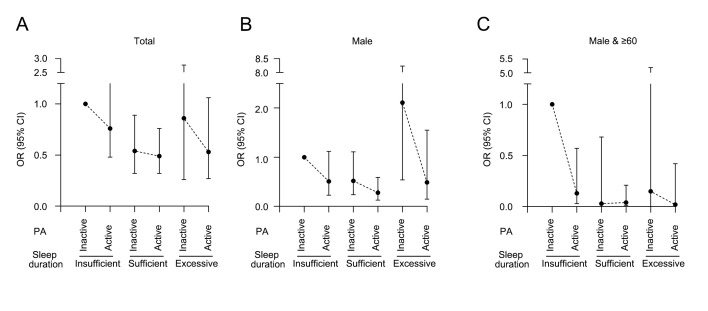
Joint association of sleep duration and PA status with constipation among participants. Active PA was always associated with a reduced constipation risk compared with inactive PA in (**Panel A**) total participants, (**Panel B**) male participants, or (**Panel C**) male participants equal to or older than 60 years old, when sleep duration was the same. All estimates accounted for complex survey designs. CI – confidence interval, OR – odds ratios, PA – physical activity.

In the joint analysis, we found participants with insufficient sleep duration and inactive PA status had the highest risk of constipation. Compared with them, sufficient sleep duration combined with either inactive PA or active PA were both found to reduce constipation risk (OR = 0.54; 95% CI = 0.32–0.89, *P* = 0.047 for inactive PA; OR = 0.49; 95% CI = 0.32–0.76, *P* = 0.015 for active PA) (**Table 3**). Besides, no matter how long the sleep duration was, the OR value of active PA status was always smaller than that of inactive PA status among all the 5590 participants (**Figure 3**, Panel A), indicating that physical activity may be linked to a comparatively stronger benefit within the same sleep group.

**Table 3:** Adjusted OR (95% CI) and *P*-value for joint association of sleep duration and PA status with constipation*

Sleep duration	PA status	OR (95% CI)	*P*-value
Insufficient	Inactive	1.00 (Reference)	
	Active	0.76 (0.48–1.21)	0.287
Sufficient	Inactive	0.54 (0.32–0.89)	0.047†
	Active	0.49 (0.32–0.76)	0.015†
Excessive	Inactive	0.86 (0.26–2.77)	0.804
	Active	0.53 (0.27–1.06)	0.114

CI – confidence interval, OR – odds ratios, PA – physical activity

*This model was adjusted for sex, age, BMI, race, education level, marriage status, PIR and drinking status.

*P < 0.05.

### Subgroup analysis

The relationship between sleep duration, PA status, and constipation was further stratified by sex. In the sex subgroup analysis, we found the relationship was stronger in male participants than in female participants. Specifically, sufficient sleep duration (OR = 0.53; 95% CI = 0.36–0.78, *P* = 0.015) and active PA (OR = 0.48; 95% CI = 0.27–0.83, *P* = 0.034) was significantly associated with lower risk of constipation. Combination of sufficient sleep duration and active PA was also related to a significantly reduced risk of constipation (OR = 0.28; 95% CI = 0.13–0.59, *P* = 0.021). The OR of combination of sufficient sleep duration and active PA was 0.28 (95% CI = 0.13–0.59, *P* = 0.021). Regardless of sleep duration, active PA was always accompanied by a lower OR value than inactive PA among 3069 male participants, which means participants with active PA were less likely to be diagnosed with constipation (**Figure 3**, Panel B). Among 2521 female participants, we only found that sufficient sleep duration was associated with lower risk of constipation (OR = 0.66; 95% CI = 0.47–0.91, *P* = 0.040). Other associations did not reach statistical significance (Table S3 in the **Online Supplementary Document**). Further, we analysed the individual and joint associations stratified by age among male and female participants. Results showed these relationships were consistent in male participants equal to or older than 60 years old. Among 677 elderly men, participants with sufficient sleep duration (OR = 0.14; 95% CI = 0.02–0.75, *P* = 0.047) had the lowest risk of constipation compared with those who had insufficient or excessive sleep duration. And individuals with active PA exhibited a decreased likelihood of constipation relative to those with inactive PA (OR = 0.27; 95% CI = 0.08–0.93, *P* = 0.050). Joint analysis found sufficient sleep duration combined with active PA significantly decreased the risk of constipation (OR = 0.04; 95% CI = 0.01–0.21, *P* = 0.006). Also, among 677 elderly men, participants with active PA generally exhibited a lower risk of constipation relative to those with inactive PA at identical sleep durations (**Figure 3**, Panel C). However, these associations were not found in male population younger than 60, nor in any age subgroup in female population (Table S4 in the **Online Supplementary Document**).

### Sensitivity analysis and PAF analysis

Previous researches have showed that depression was found related to constipation [36–38], thus, we further conducted sensitive analysis by including depression as covariates to evaluate the robustness of our primary results. The results remained similar when depression was further adjusted, that in the whole population, sufficient sleep (OR = 0.63; 95% CI = 0.49–0.82, *P* = 0.009) was associated with lower risk of constipation, while active PA was not (Table S5 in the **Online Supplementary Document**). However, combination of these two factors was still associated with lower risk of constipation (OR = 0.51; 95% CI = 0.33–0.79, *P* = 0.023) (Table S6 in the **Online Supplementary Document**). Among female participants, only sufficient sleep duration was found to reduce the risk of constipation (OR = 0.66; 95% CI = 0.48–0.92, *P* = 0.050), while in male participants, sufficient sleep duration (OR = 0.54; 95% CI = 0.37–0.81, *P* = 0.023), active PA (OR = 0.48; 95% CI = 0.27–0.85, *P* = 0.044), and combination of them (OR = 0.28; 95% CI = 0.13–0.60, *P* = 0.032) all corresponded to a lower risk of constipation (Table S7 in the **Online Supplementary Document**). Moreover, age subgroup analysis based on sex showed these results were consistent in elderly men, but not in other subgroups (Table S8 in the **Online Supplementary Document**).

To estimate the proportion of participants which could be avoided from risk of constipation if unhealthy sleep duration and/or PA deficiency were eliminated, we conducted the PAF analysis. We found that 20% and 13% of the reduced constipation was attributable to sufficient sleep duration and active PA, respectively, while 15% was attributable to the combination of sufficient sleep and active PA. For male participants, these two factors have a greater impact on constipation. Thirty-three percent of the reduced constipation were due to sufficient sleep, 57% were due to active PA, and 24% were due to both sufficient sleep and active PA. Furthermore, in male participants more than or equal to 60 years old, 57, 91, and 28% of the reduction were attributed to sufficient sleep duration, active PA, and both concurrently (Table S9 in the **Online Supplementary Document**).

## DISCUSSION

This study analysed the association between PA, sleep duration, and constipation using the NHANES data. There were three key findings. First, sufficient sleep time was independently associated with decreased odds of constipation, regardless of PA status. Second, among male participants, both sufficient sleep duration and active PA independently lowered the risk of constipation, and their combined presence was also linked to a reduced likelihood of constipation. Third, in male participants over the age of 60, sufficient sleep duration consistently related to lower OR of constipation, and those with active PA were less likely to have constipation, regardless of their sleep duration. Taken together, these results point to an important role of PA, sleep duration, and their combination in shaping constipation. We acknowledge that the findings are derived from a US-based population and may not be directly generalisable to populations in other countries or health care settings with different lifestyle patterns, dietary habits, or health care access, to some extent.

The relationship between PA and constipation remained unclear in previous studies. Several cohort studies showed that higher levels of PA were associated with a decreased risk of constipation compared with lower levels of PA (RR = 0.69; 95% CI = 0.88–0.83) and moderate levels of PA (RR = 0.87; 95% CI = 0.79–0.95) [39]. However, others found no association between functional constipation and physical activity [40]. There was also a lack of and/or discrepancies among the guidelines for recommending regular physical activity to relieve symptoms of chronic constipation [41]. These inconclusions urgently required solid and large-scale clinical studies to clarify them. Only two researches focused on the complex role of PA in constipation by using the large sample size from NHANES. Patrick B Wilson found no vigorous or no moderate recreational activity were both risk factors for constipation, which would be attenuated or insignificant after adjusted confounding factors [42]. Liu shi suggested engaging in regular recreative PA, with 20–30 minutes per session, at least five days a week, effectively reduced the likelihood of constipation [19]. However, recreational PA was only a small part of our daily PA, including working PA, transport PA, and recreational PA. Thus, suggestions based on only reactional PA were not consistent with our lifestyle, and total PA in our study could provide us more feasible guidance.

Previous researches on the relationship between sleep duration and constipation were limited. The definitions of insufficient (such as ≤4, <4.5, or <7 hours) and excessive sleep duration (such as >7, ≥8.5, ≥9, or ≥10 hours) have been variable in previous studies [8–15]. Our study adopted the current sleep consensus statements from the American Academy of Sleep Medicine and Sleep Research Society, recommending that adults should sleep at least seven hours per night on a regular basis to promote optimal health [43]. Some studies have suggested that both short and long sleep durations were associated with an increased risk of constipation [8,14], whereas others have reported an association only with insufficient sleep [9,10]. In contrast, one study found no association between sleep duration and constipation [11]. In contrast to previous studies, our findings indicate that, compared to insufficient sleep (<7 hours), sufficient sleep duration is associated with a reduced risk of constipation, whereas excessive sleep duration showed no such effect. This conclusion was more pronounced for male participants aged over 60.

The majority of studies used traditional regression models to examine the associations between sleep duration or physical activity and constipation. However, research has indicated that these two modifiable factors are codependent and require joint analyses [24,26]. These complex associations of PA and sleep likely reflect independent and joint mechanisms through which these factors are thought to influence constipation. While previous Jianfei Huang’s research has focused on the joint association of sedentary behaviour (sitting time) and sleep duration with constipation [15], the combined role of sleep duration and physical activity remains less explored. Our study reported that participants who maintained both healthy sleep duration and PA had a lower risk of constipation with a nationally representative sample. Notably, active PA was associated with lower constipation risk than inactive PA across both sleep duration groups, which may imply that PA plays a relatively prominent role in modulating constipation risk even when sleep duration is suboptimal. However, since the joint effects were analysed using combined exposure groups rather than direct head-to-head comparison of the two factors, formal statistical testing (*e.g*. interaction contrast or relative importance analysis) would be required to quantify their exact independent effect sizes. Our results underscore the combined importance of both lifestyle factors, rather than establishing a hierarchical superiority of one over the other. The lower ORs of active PA within each sleep stratum serve as supplementary evidence for the robustness of PA’s association with constipation, rather than a definitive conclusion on relative effect strength. Physical activity might act through promoting intestinal motility, prolonging colonic transit time, and improving gut microbiota [39], while dysregulated sleep duration would result in disruption in autonomic nervous system and gastrointestinal circadian rhythms [44–46].

The observed association between sufficient sleep, active physical activity, and lower constipation risk was more pronounced in elderly male participants. Several factors may contribute to this sex-specific finding. First, sex steroids (*e.g*. oestrogen in women) have been reported to facilitate gut visceromotor response, regulate gastrointestinal motility and visceral sensitivity [47–49], which may confer a certain benefit effect against constipation in women and weaken the observable association with sleep and physical activity. In contrast, elderly men experience a more gradual decline in sex hormones, potentially allowing the association of sleep and physical activity with constipation risk to be more clearly observed. Second, the intensities of physical activity may differ between sexes; we found 73.6% (n/N = 498/677) men in older age groups were in active PA status that confer greater gastrointestinal benefits, whereas only 62.5% women were in active PA status (Table S10 in the **Online Supplementary Document**). However, current study did not capture detailed physical activity subtypes, limiting our ability to explore this possibility. Third, baseline constipation prevalence may differ by sex, with 10.2% women and only 3.7% men reporting constipation (Table S11 in the **Online Supplementary Document**), potentially reducing the relative benefit of combined lifestyle factors in females due to ceiling or floor effects. Besides, these results are based on elderly man, a small sample size (677), with wide confidence intervals, which may affect the reliability of the conclusion and need to be verified in future studies with larger sample sizes of elderly males. Future studies should incorporate detailed assessments of hormonal status, subtypes and intensity of physical activity, and sex-specific baseline risk profiles to further elucidate the mechanisms underlying sex differences in the joint associations of sleep and physical activity with constipation.

In the sensitive analysis, we considered the impact of depression. This is because depression has been reported closely related to constipation, which, however, was neglected in our main analysis. For example, Judy Nee’s group found that constipation was more common in depressed individuals than non-depressed individuals and mean depression scores in patients with constipation were significantly higher than mean depression scores in healthy controls [38]. Wang Pengfei demonstrated a significant direct effect of constipation on suicidal ideation, with depression playing a partial mediating role in this interaction [37]. Besides, the risk of constipation was higher in participants with severe depression than in participants with mild depression, and further bidirectional MR analysis revealed an obviously causal effect of depression on constipation [50]. The mechanism behind this relationship was also explored. Some believed the brain-gut axis played an important role. It is a bidirectional communication network between the brain and gut, therefore, could convert emotional problems such as depression and anxiety into intestinal dysfunction [51]. Additionally, others revealed depression decreased the level of neurotransmitter serotonin (5-HT), and this could further reduce intestinal motility and epithelial growth [52]. Moreover, constipation was always thought the common side effects of antidepressants, such as tricyclic antidepressants and serotonin selective reuptake inhibitors [53].

In the PAF analysis, we found 91% reduced constipation in elderly males is attributable to active PA. This point estimate should be interpreted with caution. First, it does not imply that 91% of constipation cases were caused by inactivity; rather, it suggests that in these specific elderly male population, the observed low prevalence of constipation among active individuals translated into a high potential benefit if inactivity were eliminated. Second, we noticed that only 19.2% (n/N = 498/2597) elderly males had active PA status, far lower than other subgroups. Under such conditions, even a modest yet statistically significant association between active PA and constipation can yield a large PAF, as the formula is highly sensitive to exposure prevalence. Thus, active PA had disproportionately stronger association with constipation in elderly men subgroup. Also, the relatively wide confidence interval in this subgroup suggested limited statistical precision. This might be attributed to the restricted sample size of the elderly male subgroup (n = 677), the potential residual confounding and interindividual heterogeneity in lifestyle, physiological function, and constipation severity within this subgroup.

The strengths of our study lied in its nationally representative design, large sample size, consideration of multiple types of PA (leisure-time PA, occupation PA, and transportation PA), consentaneous sleep duration classification, and detailed differences of the conclusion among people of different genders and ages. However, there were also limitations to consider. First, this was a cross-sectional retrospective study, where the casual relationship between PA or sleep duration and constipation could not be established and the self-reported data would cause recall bias and inaccurate reporting, which could either attenuate or exaggerate the observed association between sleep duration, PA and constipation. Further prospective studies and interventions targeting lifestyle modifications are needed to elucidate the effects of different PA and sleep duration on constipation. Second, diet and medication were also vital impact factors for constipation. However, they were not included in covariations due to data limitations. It is possible that diet and medication use could modify the observed associations between sleep duration, physical activity, and constipation. Third, constipation refers to a symptom characterised by difficulty in defecation, reduced frequency of defecation, and changes in the consistency of the stool. Here, we diagnosed constipation by only consistency of the stool due to the limitation of NHANES data, making it susceptible to both underreporting and overreporting.

## CONCLUSIONS

In conclusion, our study contributes to the growing body of evidence regarding the complex association between PA, sleep duration, and constipation. We found that sufficient sleep duration and active PA were significantly linked to the reduced risk of constipation, independently or jointly. Subgroup analysis showed this finding was more pronounced in male population older than or equal to 60, but not younger adults and female participants. Given the cross-sectional nature of this study, prospective studies and interventions targeting lifestyle modifications are warranted to ascertain causality and optimal strategies for preventing and managing constipation.

## Additional material


Online Supplementary Document
