# Supplementary material for: Joint association of sleep duration and physical activity with constipation: a cross-sectional study
Source: J Glob Health. 2026 Jun 19;16:04178. doi: 10.7189/jogh.16.04178 (PMC13280867; doi:10.7189/jogh.16.04178)
Supplement: Online Supplementary Document [file jogh-16-04178-s001.pdf]

Supplement to: Zhang X, Wang Z, Zhang H, Dou W, Liu Z, Shang L. Joint association of sleep duration and physical activity with constipation: a cross-sectional study. J Glob Health. 2026;16:04178.

Supplementary file

Table S1. Outline of JoGH's Guidelines for Reporting Analyses of Big Data Repositories Open to the Public (GRABDROP) items

1. Please list all papers published by each co-author in previous three years that were based on secondary analysis of a big data repository	1. Zhang H, Xu C, Zhang J, et al. The intake of solid fat and cheese may be associated with a reduced risk of Helicobacter pylori infection status: a cross-sectional study based on NHANES 1999-2000 [J]. BMC Infect Dis. 2024, 24(1):493. doi: 10.1186/s12879-024-09392-z.<br>2. Zhang H, Wang C, Zhang J, et al. Anti-Neoplastic Effects of Coffee on Gastrointestinal Cancer as Influenced by the Dietary Background: A Cross-Sectional Study Based on NHANES 2001-2018 [J]. Cancer Med. 2026;15(2):e71612. doi: 10.1002/cam4.71612.<br>3. Zhang H, Xu C, Zhu X, et al. Associations between temporal eating patterns and energy distribution patterns with gallstones: a cross-sectional study based on NHANES 2017-2018. BMC Public Health. 2024;24(1):2994. doi: 10.1186/s12889-024-20512-x.
2. Please explain the key elements of your study design and the use of the available datasets that make your study an original scientific contribution	This study originally found that sufficient sleep as well as active PA were associated with lower risk of constipation, independently or jointly. This finding was more pronounced in male population older than or equal to 60, but not younger adults and female participants. We used National Health and Nutrition Examination Survey (NHANES[APP2.1]) 2007–2010, considering its large sample size, high-quality and representative data.

3. Please list all publications that addressed similar research questions in the same dataset and indicate where you cited them in your paper	1. Zhang G, Wang S, Ma P, et al. Association of habitual sleep duration with abnormal bowel symptoms: a cross-sectional study of the 2005-2010 national health and nutrition examination survey [J]. J Health Popul Nutr. 2024; 43(1):125. doi: 10.1186/s41043-024-00601-8. (Cited in Introduction)<br>2. Yang S, Li SZ, Guo FZ, et al. Association of sleep duration with chronic constipation among adult men and women: Findings from the National Health and Nutrition Examination Survey (2005-2010) [J]. Front Neurol. 2022; 13:903273. doi: 10.3389/fneur.2022.903273. (Cited in Introduction)<br>3. Xiong YJ, Xu HZ, Meng XD, et al. Joint association of daily sitting time and sleep duration with constipation among the US population [J]. Front Nutr. 2025; 12:1548455. doi: 10.3389/fnut.2025.1548455. (Cited in Introduction)<br>4. Yang X, Ding S, Liu S, et al. Association of Leisure-Time Physical Activity Patterns With Constipation: Evidence From the National Health and Nutrition Examination Survey [J]. Am J Gastroenterol. 2025; 120(8):1865-1869. doi: 10.14309/ajg.0000000000003498. (Cited in Introduction)<br>5. Yang H, Li B, Li H, et al. The independent and joint association between physical activity, sleep duration and daily sitting time with bone mineral density: A real world study from NHANES 2007-2018 [J]. Bone. 2024; 189:117264. doi: 10.1016/j.bone.2024.117264. Epub 2024 Sep 25. (Cited in Discussion)<br>6. Wilson PB. Associations between physical activity and constipation in adult Americans: Results from the National Health and Nutrition Examination Survey [J]. Neurogastroenterol Motil. 2020; 32(5):e13789. doi: 10.1111/nmo.13789. Epub 2020 Jan 6. (Cited in Discussion)
4. Please explain how you addressed multiple testing through an appropriately rigorous statistical threshold and indicate this in the methods section	We acknowledge the issue of multiple testing in the subgroup analysis, which is an exploratory analysis. However, after a careful literature search, we did not find examples of statistical methods for multiple testing in logistic regression models. Although as an exploratory analysis, it can provide clues for further empirical analysis, this may increase the risk of type 1 errors. Therefore, we explicitly stated in the Methods section: Also, no correction for multiple testing may increase the risk of type I error.

5. Please declare to what extent have AI chatbots been used in developing your paper and to which parts of the paper did they contribute	No AI chatbots (ChatGPT or similar) were used in the conceptualization, study design, data analysis, interpretation of results, or drafting of this manuscript.

Table S2. Characteristics of participants who were diagnosed with constipation or not.

**Characteristics**	**Constipation**		***p*-value**
	**No**	**Yes**	
**Overall**	107289198.1	8572503	
**Sex(%)**			
Female	47064145.7 (43.9)	5781051.0 (67.4)	<0.001*
Male	60225052.4 (56.1)	2791452.0 (32.6)	
**Age(median (IQR))**	42.00 (30.00,54.00)	41.00 (27.00,51.68)	0.004*
**BMI(%)**			
Normal	32543015.7 (30.4)	3189604.0 (37.3)	0.148
Underweight	1603253.3 (1.5)	121175.9 (1.4)	
Overweight	38481432.0 (36.0)	3050668.4 (35.7)	
Obese	34264327.6 (32.1)	2182660.9 (25.5)	
**Race(%)**			
Mexican American	8698353.7 (8.1)	593039.8 (6.9)	0.024*
Non-Hispanic White	76918423.8 (71.7)	5701494.7 (66.5)	
Non-Hispanic Black	10775415.6 (10.0)	1225349.2 (14.3)	
Others	10897005.0 (10.2)	1052619.3 (12.3)	
**Education level(%)**			
<High school	4341908.2 (4.1)	503944.3 (5.9)	0.013*
High school	35951287.4 (33.6)	3417547.4 (39.9)	
>High school	66816581.8 (62.4)	4651011.4 (54.3)	
**Marriage(%)**			

Married	59936720.3 (55.9)	4571141.5 (53.3)	0.113
Widowed	3244594.7 (3.0)	422022.8 (4.9)	
Divorced	9962457.7 (9.3)	964599.8 (11.3)	
Living with partner	8760778.9 (8.2)	725541.3 (8.5)	
Never married	22834330.3 (21.3)	1829275.2 (21.3)	
Separated	2529396.6 (2.4)	59922.5 (0.7)	
**PIR(%)**			
<1	18901112.0 (17.6)	1900323.2 (22.2)	0.018*
1-3	33407320.0 (31.1)	2916497.6 (34.0)	
>=3	54980766.1 (51.2)	3755682.2 (43.8)	
**Smoking(%)**			
No	60020536.5 (56.0)	4974138.1 (58.0)	0.360
Yes	47249747.1 (44.0)	3598364.9 (42.0)	
**Drinking(%)**			
No drink	9628639.0 (9.8)	1139122.1 (14.4)	0.001*
Former drinker	11006839.3 (11.2)	1393779.9 (17.6)	
Current drinker	77871948.7 (79.1)	5383779.4 (68.0)	
**Hypertension(%)**			
No	80508553.8 (75.1)	6680275.3 (78.1)	0.282
Yes	26678434.4 (24.9)	1877546.3 (21.9)	
**Diabetes(%)**			
No	100834117.2 (94.1)	8054448.2 (94.0)	0.932
Yes	6334095.8 (5.9)	518054.9 (6.0)	
**Sleep duration(%)**			
Insufficient	38672806.3 (36.0)	4048336.6 (47.2)	<0.001*
Sufficient	62522972.4 (58.3)	4001712.6 (46.7)	
Excessive	6093419.4 (5.7)	522453.8 (6.1)	

PA(%)			
Active	86716507.7 (80.8)	6413968.0 (74.8)	0.021*
Inactive	20572690.4 (19.2)	2158535.0 (25.2)	

Data shown are N (%) unless otherwise indicated.
BMI – body mass index, IQR – interquartile range, PA – physical activity, PIR – poverty-to-income ratio.
*indicates p-value < 0.05.

Table S3. Adjusted OR (95% CI) and *p*-value for association of sleep duration or PA status with constipation stratified by sex group.

		Female		Male	
		**OR (95% CI)**	***p*-value**	**OR (95% CI)**	***p*-value**
**Sleep duration**					
Insufficient		1.00(Reference)		1.00(Reference)	
Sufficient		0.66 (0.47-0.91)	0.040*	0.53 (0.36-0.78)	0.015*
Excessive		0.54 (0.28-1.06)	0.116	1.21 (0.47-3.16)	0.703
**PA status**					
Inactive		1.00(Reference)		1.00(Reference)	
Active		1.02 (0.70-1.48)	0.930	0.48 (0.27-0.83)	0.034*
**Joint analysis**					
Insufficient	Inactive	1.00(Reference)		1.00(Reference)	
	Active	0.87 (0.47-1.61)	0.681	0.51 (0.23-1.12)	0.156
Sufficient	Inactive	0.53 (0.29-0.97)	0.095	0.52 (0.24-1.11)	0.150
	Active	0.61 (0.37-1.02)	0.119	0.28 (0.13-0.59)	0.021*
Excessive	Inactive	0.46 (0.12-1.85)	0.325	2.11 (0.54-8.23)	0.330
	Active	0.5 (0.22-1.17)	0.171	0.49 (0.15-1.55)	0.280

This model was adjusted for age, BMI, race, education level, marriage status, PIR, smoking status, drinking status, hypertension and diabetes.

CI – confidence interval, OR – odds ratios, PA – physical activity.
*indicates p-value < 0.05.

Table S4. Adjusted OR (95% CI) and *p*-value for the associations of sleep duration or PA activity with constipation stratified by age among males and females.

		<45		45≤Y<60		≥60	
		**OR (95% CI)**	***P*-value**	**OR (95% CI)**	***P*-value**	**OR (95% CI)**	***P*-value**
**Male**							
**Sleep duration**							
Insufficient		1.00(Reference)		1.00(Reference)		1.00(Reference)	
Sufficient		0.61(0.36-1.02)	0.090	0.49(0.21-1.12)	0.125	0.14(0.02-0.75)	0.047*
Excessive		1.51(0.60-3.80)	0.399	1.12(0.16-7.96)	0.912	0.19(0.02-1.47)	0.146
**PA status**							
Inactive		1.00(Reference)		1.00(Reference)		1.00(Reference)	
Active		0.49(0.24-1.01)	0.084	0.34(0.08-1.41)	0.171	0.27(0.08-0.93)	0.050*
**Joint analysis**							
Insufficient	Inactive	1.00(Reference)		1.00(Reference)		1.00(Reference)	
	Active	0.49(0.19-1.31)	0.200	0.78(0.15-3.93)	0.770	0.13(0.03-0.57)	0.030*
Sufficient	Inactive	0.46(0.12-1.84)	0.312	1.59(0.26-9.71)	0.631	0.03(0-0.68)	0.063
	Active	0.32(0.12-0.86)	0.060	0.27(0.06-1.26)	0.140	0.04(0.01-0.21)	0.006*
Excessive	Inactive	3.68(0.64-21.05)	0.187	0(0-0)	0	0.15(0-5.19)	0.326
	Active	0.55(0.14-2.19)	0.422	1.6(0.12-20.99)	0.732	0.02(0-0.42)	0.038*
**Female**							
**Sleep duration**							
Insufficient		1.00(Reference)		1.00(Reference)		1.00(Reference)	
Sufficient		0.56(0.44-1.07)	0.132	0.55(0.25-1.23)	0.180	0.61(0.26-1.14)	0.140
Excessive		0.45(0.35-1.75)	0.566	0(0-0)	0	1.51(0.15-1.54)	0.244
**PA status**							
Inactive		1.00(Reference)		1.00(Reference)		1.00(Reference)	
Active		0.71(0.81-2.45)	0.255	0.71(0.27-1.83)	0.492	0.49(0.34-1.47)	0.38
**Joint analysis**							
Insufficient	Inactive	1.00(Reference)		1.00(Reference)		1.00(Reference)	
	Active	0.51(0.53-3.62)	0.526	0.76(0.17-1.52)	0.267	0.49(0.26-2.16)	0.619
Sufficient	Inactive	0.35(0.21-2.01)	0.478	0.59(0.09-1.27)	0.155	0.46(0.17-1.99)	0.422
	Active	0.36(0.38-2.48)	0.948	0.4(0.1-1.31)	0.164	0.32(0.16-1.01)	0.094
Excessive	Inactive	0.20(0.14-7.20)	0.999	0(0-0)	0	3.68(0.09-2.35)	0.387
	Active	0.31(0.27-3.85)	0.969	0(0-0)	0	0.55(0.07-1.96)	0.275

This model was adjusted for age, BMI, race, education level, marriage status, PIR, smoking status, drinking status, hypertension and diabetes.
CI – confidence interval, OR – odds ratios, PA – physical activity.
*indicates p-value < 0.05.

Table S5. Association of sleep duration and PA status with constipation (Depression adjusted).

	OR (95% CI)	*P*-value
**Sleep duration**		
Insufficient	1.00 (Reference)	
Sufficient	0.63 (0.49-0.82)	0.009*
Excessive	0.69 (0.39-1.22)	0.235
**PA status**		
Inactive	1.00 (Reference)	
Active	0.83 (0.59-1.17)	0.319

CI – confidence interval, OR – odds ratios, PA – physical activity.
*indicates p-value < 0.05.

Table S6. Adjusted OR (95% CI) and *p*-value for joint association of sleep duration and PA status with constipation (Depression adjusted).

Sleep duration	PA status	OR (95% CI)	*P*-value
Insufficient	Inactive	1.00 (Reference)	
	Active	0.76 (0.48-1.22)	0.302
Sufficient	Inactive	0.54 (0.32-0.91)	0.061
	Active	0.51 (0.33-0.79)	0.023*
Excessive	Inactive	0.82 (0.26-2.62)	0.746
	Active	0.49 (0.24-1.00)	0.098

CI – confidence interval, OR – odds ratios, PA – physical activity.
*indicates p-value < 0.05.

Table S7. Adjusted OR (95% CI) and *p*-value for association of sleep duration or PA status with constipation stratified by sex group (Depression adjusted).

		Female		Male	
		OR (95% CI)	*P*-value	OR (95% CI)	*P*-value
**Sleep duration**					
Insufficient		1.00 (Reference)		1.00 (Reference)	
Sufficient		0.66 (0.48-0.92)	0.050*	0.54 (0.37-0.81)	0.023*
Excessive		0.49 (0.24-0.97)	0.087	1.12 (0.43-2.92)	0.828
**PA status**					
Inactive		1.00 (Reference)		1.00 (Reference)	
Active		1.02 (0.70-1.50)	0.904	0.48 (0.27-0.85)	0.044*
**Joint analysis**					
Insufficient	Inactive	1.00 (Reference)		1.00 (Reference)	
	Active	0.87 (0.47-1.62)	0.688	0.51 (0.23-1.14)	0.176
Sufficient	Inactive	0.53 (0.29-0.98)	0.113	0.54 (0.26-1.13)	0.177
	Active	0.62 (0.37-1.05)	0.152	0.28 (0.13-0.60)	0.032*
Excessive	Inactive	0.45 (0.11-1.89)	0.336	1.71 (0.51-5.74)	0.432
	Active	0.44 (0.18-1.08)	0.146	0.48 (0.15-1.55)	0.287

CI – confidence interval, OR – odds ratios, PA – physical activity.
*indicates p-value < 0.05.

Table S8. Adjusted OR (95% CI) and *p*-value for the associations of sleep duration or PA activity with constipation stratified by age among males (Depression adjusted).

		<45		45≤Y<60		≥60	
		**OR (95% CI)**	***P*-value**	**OR (95% CI)**	***P*-value**	**OR (95% CI)**	***P*-value**
**Male**							
**Sleep duration**							
Insufficient		1.00 (Reference)		1.00 (Reference)		1.00 (Reference)	
Sufficient		0.62 (0.37-1.05)	0.114	0.49 (0.21-1.13)	0.132	0.13 (0.02-0.74)	0.050*
Excessive		1.32 (0.52-3.36)	0.574	1.11 (0.15-8.06)	0.917	0.18 (0.03-1.23)	0.119
**PA status**							
Inactive		1.00 (Reference)		1.00 (Reference)		1.00 (Reference)	
Active		0.51 (0.24-1.05)	0.104	0.34 (0.08-1.44)	0.179	0.26 (0.08-0.89)	0.063
**Joint analysis**							
Insufficient	Inactive	1.00 (Reference)		1.00 (Reference)		1.00 (Reference)	
	Active	0.49 (0.18-1.3)	0.200	0.78 (0.15-4.06)	0.779	0.12 (0.03-0.54)	0.031*
Sufficient	Inactive	0.49 (0.12-1.89)	0.338	1.61 (0.27-9.69)	0.624	0.03 (0-0.63)	0.066
	Active	0.32 (0.12-0.88)	0.068	0.28 (0.06-1.33)	0.160	0.04 (0.01-0.18)	0.007*
Excessive	Inactive	2.43 (0.45-13.01)	0.341	0 (0-0)	0	0.13 (0.01-2.37)	0.219
	Active	0.53 (0.13-2.10)	0.401	1.6 (0.12-21.61)	0.736	0.02 (0-0.42)	0.044*
**Female**							
**Sleep duration**							
Insufficient		1.00 (Reference)		1.00 (Reference)		1.00 (Reference)	
Sufficient		0.68 (0.44-1.05)	0.117	0.59 (0.25-1.38)	0.259	0.53 (0.26-1.09)	0.122
Excessive		0.78 (0.35-1.74)	0.558	0 (0-0)	0	0.23 (0.06-0.92)	0.071
**PA status**							
Inactive		1.00 (Reference)		1.00 (Reference)		1.00 (Reference)	
Active		1.43 (0.82-2.51)	0.240	0.71 (0.26-1.91)	0.517	0.67 (0.33-1.37)	0.306
**Joint analysis**							
Insufficient	Inactive	1.00 (Reference)		1.00 (Reference)		1.00 (Reference)	
	Active	1.39 (0.53-3.64)	0.527	0.52 (0.16-1.68)	0.319	0.74 (0.26-2.09)	0.594
Sufficient	Inactive	0.62 (0.20-1.92)	0.442	0.38 (0.09-1.54)	0.224	0.56 (0.17-1.88)	0.385
	Active	0.96 (0.37-2.47)	0.934	0.38 (0.10-1.52)	0.221	0.38 (0.15-0.97)	0.090
Excessive	Inactive	1.00 (0.14-7.20)	0.996	0 (0-0)	0	0.46 (0.10-2.24)	0.374
	Active	1.02 (0.27-3.83)	0.976	0 (0-0)	0	0.08 (0.01-0.81)	0.077

CI – confidence interval, OR – odds ratios, PA – physical activity.
*indicates p-value < 0.05.

Table S9. PAF analysis of sleep duration and PA status with constipation among participants.

	Total		Male		Male & ≥60	
	**PAF**	**SE**	**PAF**	**SE**	**PAF**	**SE**

**Sleep duration**						
Sufficient	-0.2	0.05	-0.33	0.08	-0.57	0.27
**PA status**						
Active	-0.13	0.09	-0.57	0.24	-0.91	0.49
**Joint analysis**						
Sufficient+Active	-0.15	0.04	-0.24	0.07	-0.28	0.15

PA – physical activity, PAF analysis – population attributable fraction analysis, SE – standard error.

Table S10. PA levels in different subgroups.

Subgroups		Active PA (%)	Inactive PA (%)	*p*-value
Male	<45	1429 (88.8)	180 (11.2)	<0.001
	45≤Y<60	670 (85.6)	113 (14.4)	
	≥60	498 (73.6)	179 (26.4)	
Female	<45	989 (77.7)	284 (22.3)	<0.001
	45≤Y<60	456 (70.2)	194 (29.8)	
	≥60	374 (62.5)	224 (37.5)	

PA – physical activity.

Table S11. Prevalence of constipation and PA status in elderly population.

		Female (%)	Male (%)	*p*-value
Constipation	Yes	61 (10.2)	25 (3.7)	<0.001
	No	537 (89.8)	652 (96.3)	
PA status	Active	374 (62.5)	498 (73.6)	<0.001
	Inactive	224 (37.5)	179 (26.4)	

PA – physical activity.

STROBE Statement—Checklist of items that should be included in reports of ***cross-sectional studies***

	Item No	Recommendation
**Title and abstract**	1	(*a*) Indicate the study's design with a commonly used term in the title or the abstract
		(*b*) Provide in the abstract an informative and balanced summary of what was done and what was found
**Introduction**		
Background/rationale	2	Explain the scientific background and rationale for the investigation being reported
Objectives	3	State specific objectives, including any prespecified hypotheses
**Methods**		
Study design	4	Present key elements of study design early in the paper
Setting	5	Describe the setting, locations, and relevant dates, including periods of recruitment, exposure, follow-up, and data collection
Participants	6	(*a*) Give the eligibility criteria, and the sources and methods of selection of participants
Variables	7	Clearly define all outcomes, exposures, predictors, potential confounders, and effect modifiers. Give diagnostic criteria, if applicable
Data sources/ measurement	8*	For each variable of interest, give sources of data and details of methods of assessment (measurement). Describe comparability of assessment methods if there is more than one group
Bias	9	Describe any efforts to address potential sources of bias
Study size	10	Explain how the study size was arrived at
Quantitative variables	11	Explain how quantitative variables were handled in the analyses. If applicable, describe which groupings were chosen and why
Statistical methods	12	(*a*) Describe all statistical methods, including

		those used to control for confounding
		(*b*) Describe any methods used to examine subgroups and interactions
		(*c*) Explain how missing data were addressed
		(*d*) If applicable, describe analytical methods taking account of sampling strategy
		(*e*) Describe any sensitivity analyses
**Results**		
Participants	13*	(a) Report numbers of individuals at each stage of study—eg numbers potentially eligible, examined for eligibility, confirmed eligible, included in the study, completing follow-up, and analysed
		(b) Give reasons for non-participation at each stage
		(c) Consider use of a flow diagram
Descriptive data	14*	(a) Give characteristics of study participants (eg demographic, clinical, social) and information on exposures and potential confounders
		(b) Indicate number of participants with missing data for each variable of interest
Outcome data	15*	Report numbers of outcome events or summary measures
Main results	16	(*a*) Give unadjusted estimates and, if applicable, confounder-adjusted estimates and their precision (eg, 95% confidence interval). Make clear which confounders were adjusted for and why they were included
		(*b*) Report category boundaries when continuous variables were categorized
		(*c*) If relevant, consider translating estimates of relative risk into absolute risk for a meaningful time period
Other analyses	17	Report other analyses done—eg analyses of subgroups and interactions, and sensitivity analyses
**Discussion**		
Key results	18	Summarise key results with reference to study

		objectives
Limitations	19	Discuss limitations of the study, taking into account sources of potential bias or imprecision. Discuss both direction and magnitude of any potential bias
Interpretation	20	Give a cautious overall interpretation of results considering objectives, limitations, multiplicity of analyses, results from similar studies, and other relevant evidence
Generalisability	21	Discuss the generalisability (external validity) of the study results
**Other information**		
Funding	22	Give the source of funding and the role of the funders for the present study and, if applicable, for the original study on which the present article is based

*Give information separately for exposed and unexposed groups.

Note: An Explanation and Elaboration article discusses each checklist item and gives methodological background and published examples of transparent reporting. The STROBE checklist is best used in conjunction with this article (freely available on the Web sites of PLoS Medicine at http://www.plosmedicine.org/, Annals of Internal Medicine at http://www.annals.org/, and Epidemiology at http://www.epidem.com/). Information on the STROBE Initiative is available at www.strobe-statement.org.
